# Effect of orthodontic forced eruption for implant site development in the maxillary esthetic zone: A systematic review of clinical data

**DOI:** 10.34172/japid.2024.022

**Published:** 2024-10-07

**Authors:** Mohammadreza Talebi Ardakani, Aida Kheiri, Majid Torabzadeh, Amirhosein Mahmoudian, Mohammad Hossien Talebi, Amir Talebi

**Affiliations:** ^1^Department of Periodontics, School of Dentistry, Shahid Beheshti University of Medical Sciences, Tehran, Iran; ^2^Queen’s Belfast Dental School, Belfast, Northern Ireland; ^3^Dental School, Universidad Europea de Madrid, Madrid, Spain

**Keywords:** Bone regeneration, Dental implant, Orthodontic extrusion

## Abstract

Dental implant placement in the esthetic zone is associated with challenges for clinicians. The best esthetic outcome of this procedure can be obtained through precise management of hard and soft tissue. Orthodontic forced eruption (OFE) has presented an alternative approach to augment hard and soft tissues, which can be applied rapidly or slowly. OFE of hopeless teeth with its periodontal attachment results in a favorable implant preparation site. Therefore, the present systematic review evaluated the effect of implant site preparation using OFE in hopeless teeth. A complete electronic search was performed in PubMed/MEDLINE, Scopus, and Google Scholar from June 2020 to November 2023. The search was limited to clinical English language studies. Studies were excluded if OFE was performed without implant placement. Finally, 15 studies with a total of 21 teeth, all located in the maxillary anterior region, were included in this study. In eight studies, bone grafting procedures were performed before implant placement. Using OFE could rapidly prepare the implant site by enhancing hard and soft tissues. However, additional interventions like guided bone regeneration should be considered case-dependent.

## Introduction

 Using dental implants to address esthetic zones brought a new challenge for clinicians. The effective osseointegration of the implant, harmony between the final restoration and the neighboring teeth, and the health of the surrounding soft and hard tissues are the aims of such restoration.^1^

 Four factors should be addressed when evaluating an implant site in an esthetic zone: smile line, soft-tissue morphology, tooth morphology, and osseous architecture.^2^ The placement of an implant in an appropriate connection to the intended restoration is critical for the best esthetic and functional outcomes, which would require sufficient alveolar bone volume and position.^3-5^ Various surgical methods, such as distraction osteogenesis,^6^ guided tissue regeneration,^7^ and graft procedures, have been suggested to maintain or repair the alveolar ridge.^8^ These techniques can be used to treat the ridge defect at the time of extraction or later. Orthodontic forced eruption (OFE) is another method of soft and hard tissue augmentation. According to Heithersay^9^ and Ingber,^10^ orthodontic treatment causes the periodontal ligament to be pulled during eruption, which increases bone volume and causes osteoblastic activity to occur where the periodontal attachment is located.^11^ The gingiva and bone linked by the periodontal ligaments migrate in the same direction of the tooth’s coronal movement.^12^ Additionally, this method may be used to move the root, creating room and anchoring for an implant. In 1993, Salama and Salama^13^ suggested modifying the forced eruption method. By forced orthodontic extrusion of “hopeless” teeth and their periodontal structures, this novel technique, known as “orthodontic extrusive remodeling,” was employed to improve the soft and hard tissue profiles of possible implant sites.^13^ Hence, the present study aimed to assess the effect of site preparation of dental implants using OFE in hopeless teeth.

## Methods

 This systematic review was conducted according to the PRISMA (Preferred Reporting Items for Systemic Reviews and Meta-Analyses) guidelines statement.

###  Focused question

 The following focused question was formulated to outline the search strategy: *“What is the effect of orthodontic extrusion on site development for implant placement in the esthetic zone?”* with “site development” referring to the optimization of the implant spatial positioning and minimizing the need for adjunctive regenerative treatments.

###  Search strategy

 A search of electronic databases, including Scopus, PubMed/MEDLINE, and Google Scholar, was performed from January 2000 to November 2023. The search was limited to English-language studies with available full texts. Furthermore, a hand search was conducted to assess the studies that were not electronically available. The search was performed based on the following keywords: [orthodontic AND extrusion] AND [dental AND implant], [orthodontic AND extrusion] AND [site AND development], [orthodontic AND extrusion] AND [dental AND implant] AND/OR [[soft OR hard] AND tissue AND [regeneration OR augmentation]].

###  Selection criteria

 All the clinical studies that used orthodontic extrusion for further implant placement in the esthetic zone were included. Abstracts, letters, and reviews were excluded. Studies were excluded if orthodontic extrusion was performed solely or adjacent to a dental implant. De-duplicating was done manually, and finally, eligible studies were included, and their full texts were obtained.

 Two independent reviewers assessed the full texts, and the following data were extracted and further classified in a table: type of study, number of patients, gender, age, number of tooth/teeth, type of jaw (maxilla/ mandible), orthodontic movement procedure, implant characteristics, follow-up, and outcome. Any disagreement between reviewers was resolved following discussion.

## Results

**Figure 1:**
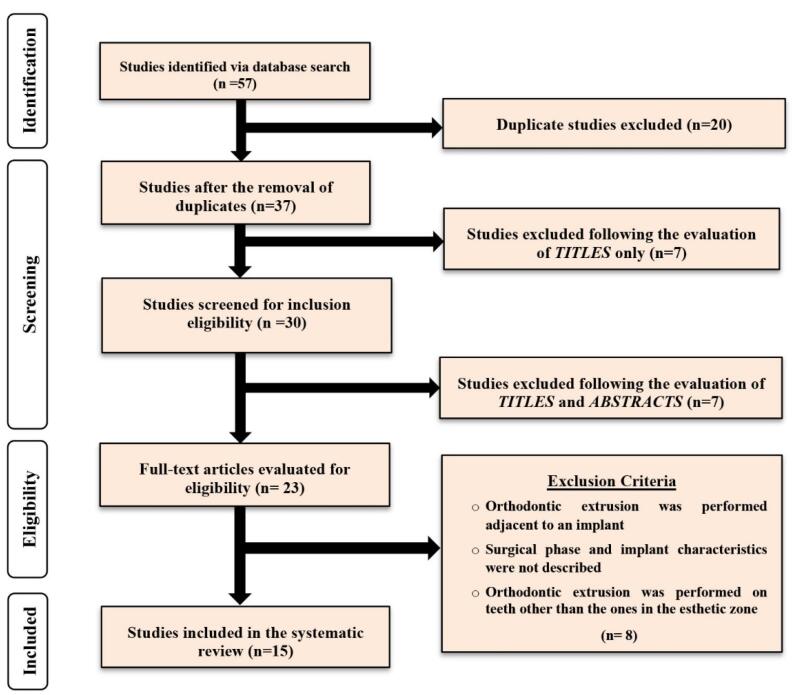
Search strategy flowchart

 Initially, 57 articles were identified via a search through the above-mentioned databases. After removing duplicate investigations, 37 studies were further considered. After evaluating titles, 7 studies, and after assessing the titles with their corresponding abstracts, 7 more studies were excluded. The full texts of the 23 remaining studies were evaluated based on the predetermined inclusion/exclusion criteria; consequently, 15 studies were included in the current systematic review. Figure 1 shows the strategy flowchart of the present investigation.

###  Study characteristics

 All the included studies were case reports. Sixteen individuals have been documented in the literature, where orthodontic extrusion was used to establish implant sites in the esthetic zone. Patients’ age varied from 22 to 57 years and consisted of 11 women and 5 men. A total of 21 teeth underwent extrusion and further extraction and implant placement. All these teeth were maxillary anteriors, and central incisors were the most common.

 The orthodontic extrusion period varied noticeably between studies, from the shortest period of 3 months^14^ to the longest one of 12 months.^15^ In two studies, implant insertion was performed 6^15^ and 7^16^ months after tooth extraction, and in 8 studies, an additional bone grafting procedure was performed before or simultaneous with implant placement.^14,16-22^ The most chosen implant diameter was 3.75 × 11 mm in two studies.^23,24^

 Among 8 studies mentioning follow-up periods, the shortest time was 11.5 months,^19^ and the longest period was 10 years.^16^ Table 1 shows more details about the included studies.

**Table 1:** Studies that used OFE in hopeless teeth before implant placement

**Author/year**	**Type of study**	**No. of patients/** **Gender/age (mean age)**	**Tooth/teeth** **Max/Man**	**Orthodontic movement procedure**	**Implant characteristics**	**Follow up**	**Outcome**
Maiorana et al, 2012^14^	Case report	1/ F/42 years old	2 maxillary central incisors	Slow orthodontic extrusion for 3 mon.	2 Astra® dental implants positioned following a prosthetic guide + bovine deproteinized bone (Bio-Oss Geistlich CH®) for ridge reconstruction + two resorbable membranes + two provisional crowns + connective tissue periosteal flap after 3 mon	2 years	1. Good amount of keratinized mucosa and 2. Signs of osseointegration of the dental implants in radiographic investigation.
Hasson and Hasson, 2016^15^	Case report	1/ F/27 years old	Maxillary right central and lateral incisor	1. Orthodontic therapy for 12 mon (discontinued when no further movement could be achieved). 2. Extraction of #7 and #8 3. socket filling with xenograft	After 6 mon: Implant positioning in a slightly palatal direction	NM	Stable gingival level and papillae A small gingival crevice-invagination on the distal aspect of tooth #8 due to excess epithelial tissue removal at the time of gingival grafting
Correia et al, 2022^16^	Case report	1/ F/ 24 years old	Maxillary right central incisor	1. Slow traction (light force of 15 g) for 3 mon 2. Stabilization period of 4 mon.	7 mon after beginning slow orthodontic: 1. Extraction socket preservation executed with a xenograft covered with a connective tissue graft collected from the palate. 2. After 4 mon, 3.75 x 10 mm; Osseotite, Zimmer Biomet + GBR using a xenograft and collagen membrane 3. 4 mon after implant placement: connective tissue graft	1 year	NM
Paolone et al, 2008^18^	Case report	1/ M/ 57 years old	Maxillary left central incisor	1. Slow and light osseous movement of 0.5 mm/mon using lingual brackets 2. Brackets re-bonded apically 2 mon later 3. stabilization before extraction and implantation for 6 mon	1. Atruamatic extraction using periotome 2. Implant placement (A FRIALIT-2 4.5/15 mm (Dentsply FRIADENT®) root-form) + implant submerge for atraumatic healing. 3. Definitive restoration after 12 mon.	NM	NM
Maeda and Sasaki, 2015^19^	Case report	1/ M / 28 years old	Maxillary right central incisor	Brackets + sectional arch wire with anchorage on #12, #21 with light force (30~50 g) in an incisal direction for 3 mon + mon m of retention.	Root form type implant (Osseotite Implant 415, 3i) (4 × 15 mm) + deproteinized cancellous bovine xenograft particles + enamel matrix derivative (Emdogain, Biora) + SCTG + dissolvable collagen membrane	11.5 mon	1. An esthetic implant-supported crown with symmetric soft tissue contours was achieved with the combined orthodontic extrusion, orthodontic alignment, and grafting procedures. 2. The maintenance phase has been uneventful.
Watanabe et al, 2013^20^	Case report	1/ M/ 41 years old	Maxillary right central incisor	Extrusion for 5 mon using brackets and cobalt chromium wire (coronal movement of approximately 6 or 7 mm at final recall).	1. Atraumatic extraction 2.5 mon after completion of extrusion 2.3.75 × 15 mm implant Nobel Mk III Nobel Biocare was placed immediately after extraction + GBR 3. The definitive restoration was delivered 6 mon later.	4 years	1. Excellent long-term prognosis for the restoration. 2. Continuing presence of adequate labial bone.
Rokn et al, 2012^21^	Case report	1/ F/ 34 years old	Maxillary right and left central and lateral incisors	1. Slow orthodontic extrusion for 4 m using edgewise brackets bonded to the surfaces of all maxillary teeth between and including the first molars. 2. Additional 4 mon of stabilization	1. Extraction of 4 incisors + sockets filling with bone grafting material (Bio-Oss) up to the level of the crestal bone. 2. two implants (3.5 mm x 15 mm), in the lateral incisor sites using the template with the flapless method. 3. The definitive prosthesis was delivered to the patient after 4 mon.	NM	NM
Caberlotto et al. 2018^22^	Case report	1/ F/ 50 years old	Maxillary left lateral incisor	The right canine to the left canine were ligated with a stainless steel archwire to anchor a segmented NiTi archwire attached to the left second incisor (4 mon)	3.5x12 mm implant (CLC Conic, CLC Scientific vicenza, Italy + GBR procedure using xenograft + a resorbable collagen membrane.	2 years	1. No significant changes in soft tissue contours 2. Radiographic examination highlighted maintained bone levels around the implant platform
Chambrone and Chambrone, 2005^23^	Case report	1/ M/ 48 years old	maxillary right lateral incisor	Slow orthodontic extrusion for 10 wk and followed by 10 wk of stabilization	(3.75 mm × 11 mm implant, Osseotite, 3i, Palm Beach Gardens, Fla, and remained unloaded for 6 m.	NM	1. Increase in the zone of the attached gingiva. 2. Satisfactory emergence profile for the dental implant.
Kim et al, 2011^24^	Case report	1 /F/ 30 years old	Maxillary right central incisor	1. Endodontic treatment 2. Fabrication of an acrylic resin interocclusal appliance 3. Slow orthodontic tooth extrusion of the right lateral and central incisors (70-100 g for 6 mon)	3.75 mm x 11 mm screw-type (Neodent) + Definitive metal ceramic crown	5 years	1. Esthetic improvement and soft tissue stability compared to the preoperative condition. 2. Improvement of the alveolar bone around the implant and bone formation in radiographic images.
Holst et al, 2007^25^	Clinical report	1/ F/ 23 years old	left maxillary central incisor	Extrusion via ligation of an elastic archwire (Sentalloy 0.14) Following a 4-w extrusion period, stabilization of the extruded tooth for 12 wk.	4 x 13-mm implant (NobelReplace RP; Nobel Biocare AB,	NM	Predictable clinical outcomes in using multidisciplinary treatment approaches combining OFE, immediate implant placement, and immediate provisional restoration protocols
de Molon et al, 2013^26^	Case report	1/ F/ 22 years old	Maxillary right lateral incisor	1. 12 wk of orthodontic extrusion 2. Stabilization for 4 mon	3.3 mm × 11 mm implant (Neodent, Curitiba, Brazil)	5 years	Prior to extraction: observation of clinical, and radiographically, a substantial interproximal and vertical bone formation and an increase in the amount of attached gingiva.
Joo et al, 2016^27^	Case report	1/ F/ 46 years old	Maxillary right canine	A light force of approximately 10 to 15 g, and the rate of the force for approximately 1.0 mm/mon. 8 months of eruption and 2 mon for stabilization	4.0 mm x 11.5 mm (Branemark MK III Groovy, Nobel Biocare)	NM	1. Esthetic improvement of the implant 2. Improvement of the alveolar bone around the implant in radiographic images.
Paolone et al, 2018^28^	Case series	2.1/F/ 57 years old	Maxillary left central incisor	1. Lingual appliance was applied only on the upper anterior teeth, including the first premolars. 2. Stabilization for 6 mon.	1. 4.5x15 mm (Dentsply FRIADENT) root-form fixture. 2. After 6 mon, the second surgical phase was performed. 3. After 12 mon, a definitive prosthesis was obtained.	NM	NM
		2.1/F/ 41 years old	Maxillary left central incisor	1. full mouth bonding on the upper teeth with lingual brackets + a sectional extrusive set up in indirect lingual bonding on the 21 (1 mon for each mm of extrusion)	1. 4.5/15 mm (Dentsply FRIADENT), the osseous defect was filled with autologous particulate bone and further covered with Titanium-reinforced e-PTFE Membrane. 2. After 12 mon, membrane removal + vestibular graft was performed. 3. After 4 mon, a temporary crown was obtained.	NM	NM

F: female; GBR: guided bone regeneration; Mon: month(s); SCTG: subepithelial connective tissue graft; NM: not mentioned; PES: pink esthetic score; wk: week(s).

## Discussion

 Dental implant placement has become the gold standard treatment for replacing missing teeth for a long time.^29,30^ Bone and tissue loss following inflammatory disease in periodontium could lead to departure from normal alveolar morphology and further difficulties for implant placement where hopeless teeth exist.^24,31^ OFE has been recommended as the only non-surgical adjunctive way to enhance hard and soft tissue conditions for implant placement.^31,32^ Hence, the present review evaluated the effect of OFE for implant placement in the esthetic zone.

 Almost all the 15 studies included in our review reported positive outcomes of OFE from clinical, radiographic, and esthetic aspects. Continuous light force was the preferred approach in most of the studies. According to Kim and colleagues’ study, this light force could induce the stretch of gingival and periodontal fibers and further formation of new bone and gingiva in the coronal part.^24^

 In multiple studies, noticeable advantages of OFE for hopeless teeth before implant placement were mentioned: first, the hopeless teeth could aid in oral rehabilitation procedures. Second, the patient’s discomfort would decrease since the hopeless teeth solve esthetic issues, and third, by following this treatment, periodontal ligaments’ capacity as a distraction osteogenesis means would be used by stimulation of biological potential of periodontal ligament and modifying the morphology of an intrabony defect to a desirable one.^11,28,33,34^

 However, this treatment modality is not without complications; esthetic problems due to the presence of wires and brackets, phonetic discomfort, difficulty in orthodontic force control, undesirable external root resorption of adjacent teeth, gingival recession, and buccal bone dehiscence could be considered the drawbacks of the above-mentioned technique.^24,28^ As was observed in Kim and colleagues’ study, the radiographic evaluation revealed apical root resorption in the lateral incisor adjacent to the targeted tooth. This phenomenon mainly contributed to a greater magnitude of force than intended.^24^

 On the other hand, OFE has not always provided enough bone, so further surgical regenerative procedures were needed in multiple studies^14,16,17,19-22,28^ to provide acceptable hard tissue morphology.

 To precisely perform case selection, good plaque control, resolution of periodontitis, presence of at least 1/3 to 1/4 of apical attachment, and feasibility of adequate stabilization period must be considered.^35^

 Magnitude, duration, and retention period varied in the studies. In Joo’s study, 1 mm/month of movement had similar results to 1 mm/week of coronal displacement.^27^ However, Isola’s study emphasized using low and controlled ( < 100 g) forces to obtain < 1 mm/month movement.^33^ The retention period was reported to be 6‒12 weeks,^27^ while in Alsahhaf and Att’s study, a minimum of 3‒6 months was suggested.^30^ This variation in OFE procedures necessitates careful examination and individualized treatment planning.

 The current review has its limitations. First, the number of available studies in the literature is limited, which does not allow for a comprehensive conclusion to be drawn. The lack of a long-term follow-up negatively affects the reliability of the outcomes. Moreover, all the studies included in this review are case reports or case series that will negatively affect the quality of the generated evidence, and the interpretation of the results should be carried out with caution.

## Conclusion

 In conclusion, OFE of hopeless teeth seems an acceptable alternative to enhance soft and hard tissue conditions for future implant placement. Nevertheless, additional interventions such as submerged healing and guided bone regeneration should be considered in special cases to achieve the best outcome in the esthetic zone.

## Competing Interests

 The authors of the current manuscript declare no conflicts of interest regarding the publication of the presented paper.

## Data Availability Statement

 The datasets used and/or analyzed during the current study are available from the corresponding author upon reasonable request.

## Ethical Approval

 Not applicable.
